# A compact external-rail linear actuation mechanism with a high stroke-to-length ratio for wearable exoskeleton applications

**DOI:** 10.3389/frobt.2026.1843206

**Published:** 2026-08-12

**Authors:** Tianmai Sun, Filip Stefanovic

**Affiliations:** Department of Biomedical Engineering, University at Buffalo, Buffalo, NY, United States

**Keywords:** compact exoskeleton, assistive robotics, compliant joint, linear actuator, tendon drive

## Abstract

**Introduction:**

Tendon-driven exoskeletons are often limited by mechanical bulk, complex cable routing, and large externally mounted actuators. Similarly, conventional linear actuators used in wearable exoskeleton systems are often limited by low stroke-to-body-length ratios that restrict the range of motion. This study presents the mechanical design of a novel Rail-guided Inline DirEct dRive (RIDER) with a high stroke-to-length ratio for wearable exoskeletons.

**Methods:**

The proposed system creates a linearized RIDER system using a “motor-driven cart” layout that travels along a toothed twin-rail structure. The prototype system was developed using 3D-printed materials and open-source electronics, and the resultant force–velocity performance was experimentally evaluated under vertical loading. Vertical displacement was measured using an ultrasonic sensor (HC SR-04), and the corresponding velocity was computed via a finite-difference approximation.

**Results:**

The actuator demonstrated a maximum free-load velocity of 7.27 cm/s and a maximum force output of 103.7 N prior to slipping. When integrated into a tendon-driven system, the duo configuration produced a 6.22 Nm torque output with a range of motion of 120° or a 10.37 Nm torque output with a range of motion of 72°. The actuator also demonstrates a 190% (i.e., 0.58 vs 0.2) higher stroke-to-length ratio than a representative telescopic actuator of similar geometry.

**Conclusion:**

In this study, we show that the RIDER system demonstrates a wearable, rail-based actuation strategy capable of adjustable torque amplification and improved spatial-efficiency integration for human–robot interaction in wearable robotics. The current study focuses on the benchtop mechanical validation of the RIDER system as a proof-of-concept for future integration into wearable exoskeletons. This research is part of a larger study to develop adaptive exoskeletons that enable scalable performance improvements for assistive robotics in the workplace and neurorehabilitation.

## 1 Introduction

A fundamental challenge in wearable exoskeletons is the spatial configuration of the actuator systems (Masud et al., 2023; Urrea and Agramonte, 2023; Di Natali et al., 2020). For example, compact and lightweight actuation systems with high power-to-mass ratios are required to reduce distal inertia and improve dynamic performance during both rehabilitative assistance and performance augmentation (Dollar and Herr, 2008). In upper-limb exoskeletons, excessive actuator mass increases the rotational inertia and interferes with natural movement (Vantilt et al., 2019). Accordingly, the development of actuation architectures capable of producing sufficient force output while maintaining low structural mass remains an important research focus. Among these actuator architectures, tendon-driven cable transmission systems have broad utility in wearable robotics due to their force transmission (Li et al., 2022; Tan et al., 2024), low backlash, and inherent back drivability (Perry et al., 2007). Such architectures offer improved biomechanical compatibility and flexibility in routing assistive forces across joints. However, many upper-limb exoskeletons have challenges with mechanical bulk (Zhao et al., 2023) and complex cable routing (Bardi et al., 2022), which can limit portability and wearable integration (Perry et al., 2007). For example, the proximal placement of actuators and bearings can reduce the system’s inertial effects but can cause interference during natural arm motion. Furthermore, the strength-to-weight limitations of system actuation hardware have historically required power supplies and control components to be externally mounted, limiting mobility (Morris et al., 2023; Khawaja et al., 2025). For example, the tendon-driven cable exoskeleton design presented by Perry et al. (2007) showed a high torque-to-weight output (2.2 Nm/kg) but was bulky (41.3 kg) and thus needed to be mounted to a stationary base for weight support. Similarly, the HAL-3 developed by Kawamoto and Sankai (2002) produced high torques (32 Nm) but required an external backpack to house the electronic components, such as the controller unit and motor driver. Finally, in the case of pneumatic-actuated active orthoses developed by the University of Michigan, a high torque (70 Nm) was produced at the ankle joint for plantar flexion. However, the trade-off was the use of a stationary compressor for pneumatic actuation, which limited the user’s mobility (Ferris et al., 2005). Thus, there is a critical need to develop more scalable systems for wearable exoskeletons.

Hydraulic and electrical linear actuators have been employed in various lower-limb exoskeleton designs due to their high force output capabilities, compact size, and lightweight (Zoss et al., 2006). However, such actuators are often characterized by limited geometric flexibility and limitations to limb extension motions (Siviy et al., 2023). Functional limitations to a user’s range of motion can complicate integration into portable upper-limb wearable platforms and thus limit utility (Pires et al., 2025). For example, Zoss et al. (2006) developed the BLEEX system, where the range of motion (ROM) was limited due to the linear actuator’s spatial orientation. Often, these systems are oriented in this way to maximize the force or torque output at the expense of ROM. Stronger actuators can be used as alternatives, such as hydraulic systems, to increase power output. However, hydraulic actuators can leak fluid and are heavy, which is counterproductive when integrated into body-worn exoskeletons (Spong, 2012). Other options include telescoping linear actuators that can be electronically driven but can also be bulky and can cause unnatural biomechanics in the user if unproperly mounted. Regardless, these can be well suited for some applications such as in the J_Exo system developed for gait assistance (Ju et al., 2024). Therefore, larger travel distances were proportional to larger actuator envelopes, causing some limitations in compact application. These configurations have a profound effect in portable systems, such as arm exoskeletons, due to the limited space on the upper arm and the associated inertial effects.

While previous tendon-driven and wearable actuator systems have explored placement, cable routing, and spatial efficiency, these characteristics are often explored independently. For example, the CADEN-7 system optimized distal inertia by reconfiguring the cable pulley while remotely placing motors to reduce the weight (Perry et al., 2007). Conversely, the CAREX-7 system mounted the motors directly on the platform, limiting portability (Cui et al., 2017). Another system, known as the Bowden tendon-driven system, used a lightweight and compact actuator, but the pulling direction was perpendicular to the actuator length, limiting the torque output. It additionally complicated the cable routing and placement of the actuator (Wei et al., 2018), leading to functional limitations such as range of motion. Thus, there is a need to develop new tendon-driven mechanisms so that the weight and cabling configurations can improve multidimensional functionality. As such, the goal of this study is to develop a novel Rail-guided Inline DirEct dRive (RIDER) actuation mechanism system for the tendon-based drive of exoskeletons. The RIDER will have a longer stroke-to-length ratio, simpler cable routing, and compact geometry when compared with other actuation systems.

In this study, we propose a novel rail-based linear actuation system for tendon-driven exoskeletons. The scope of this study evaluates the mechanical performance of the RIDER system (such as torque and force output) and demonstrates how it would be integrated into an exoskeleton. It consists of a twin-rail-based actuator that transmits force via a routed cable across a pulley system that connects to the forearm segment. This configuration preserves the biomechanical benefits of tendon-driven systems while improving portability and structural integration within a mobile exoskeleton. Here, we focus on the benchtop mechanical validation of the actuator architecture under controlled loading conditions for future integration into wearable exoskeletons. The work in this project is part of a larger project to develop accessible adaptive assistive exoskeletons for the workplace and neurorehabilitation.

## 2 Materials and methods

This section provides a system overview of how the RIDER system is designed, including hardware, software, and electronics. Then, the theoretical torque and force requirements for the system are defined as would be required for use in future exoskeletons. Then, an overview of the experimental protocol is provided to test the RIDER system.

### 2.1 System overview

**FIGURE 1:**
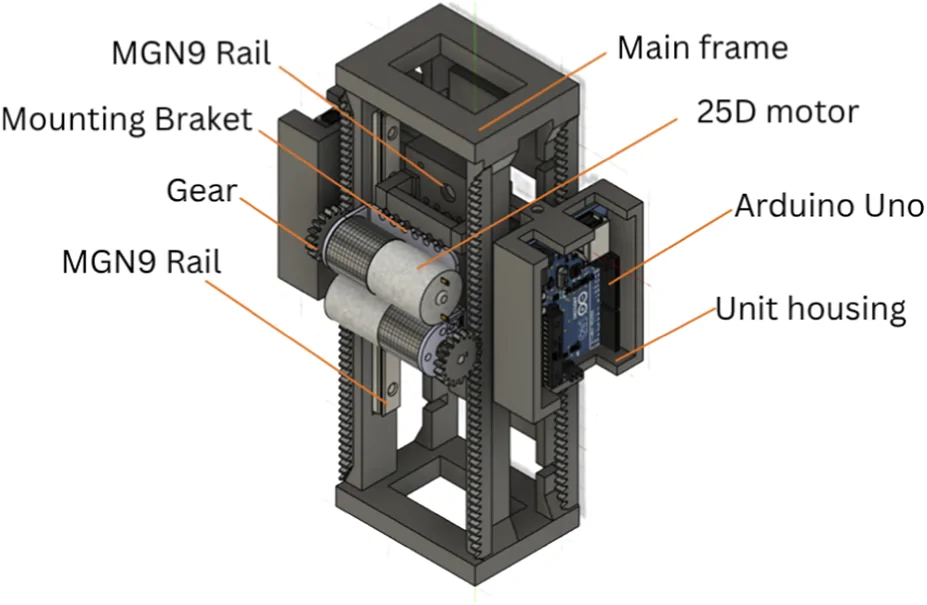
3D model of the RIDER system with parts labeled.

In contrast to previous upper-limb exoskeletons, our proposed attachment configuration optimizes longitudinal space utilization by aligning the rail-based actuator along the upper arm’s longitudinal axis (Figure 1). We hypothesize that this design improves geometric integration and enhances wearability.

### 2.2 Operating design principle

**FIGURE 2:**
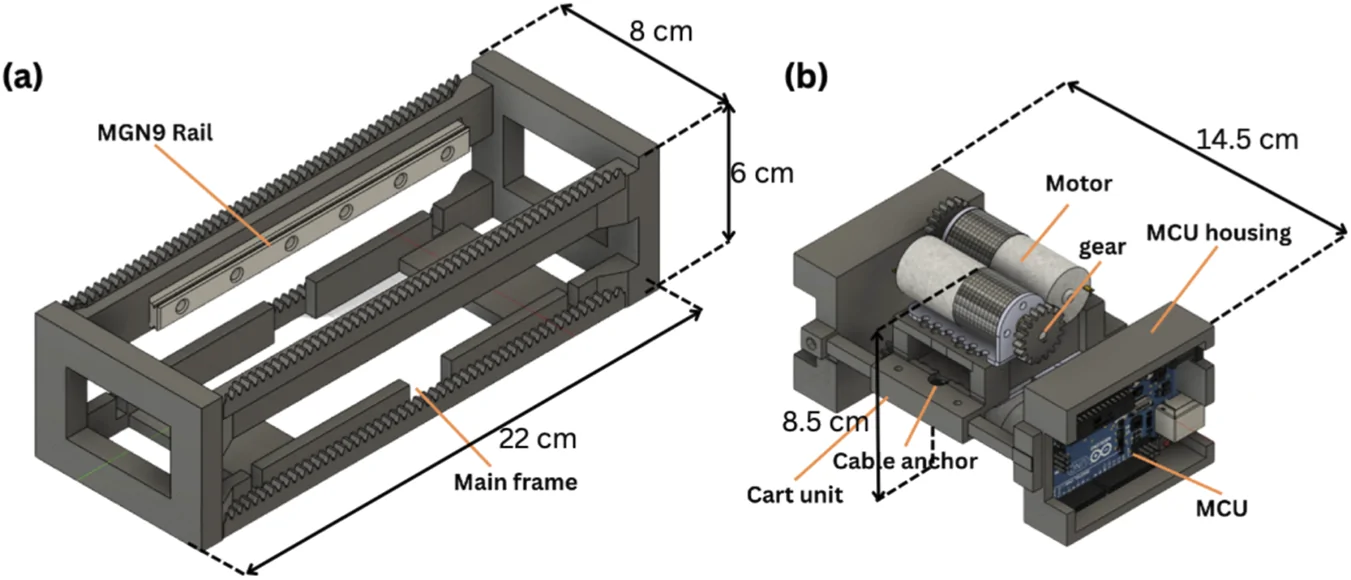
**(a)** Main frame and **(b)** cart unit design configuration.

**FIGURE 3:**
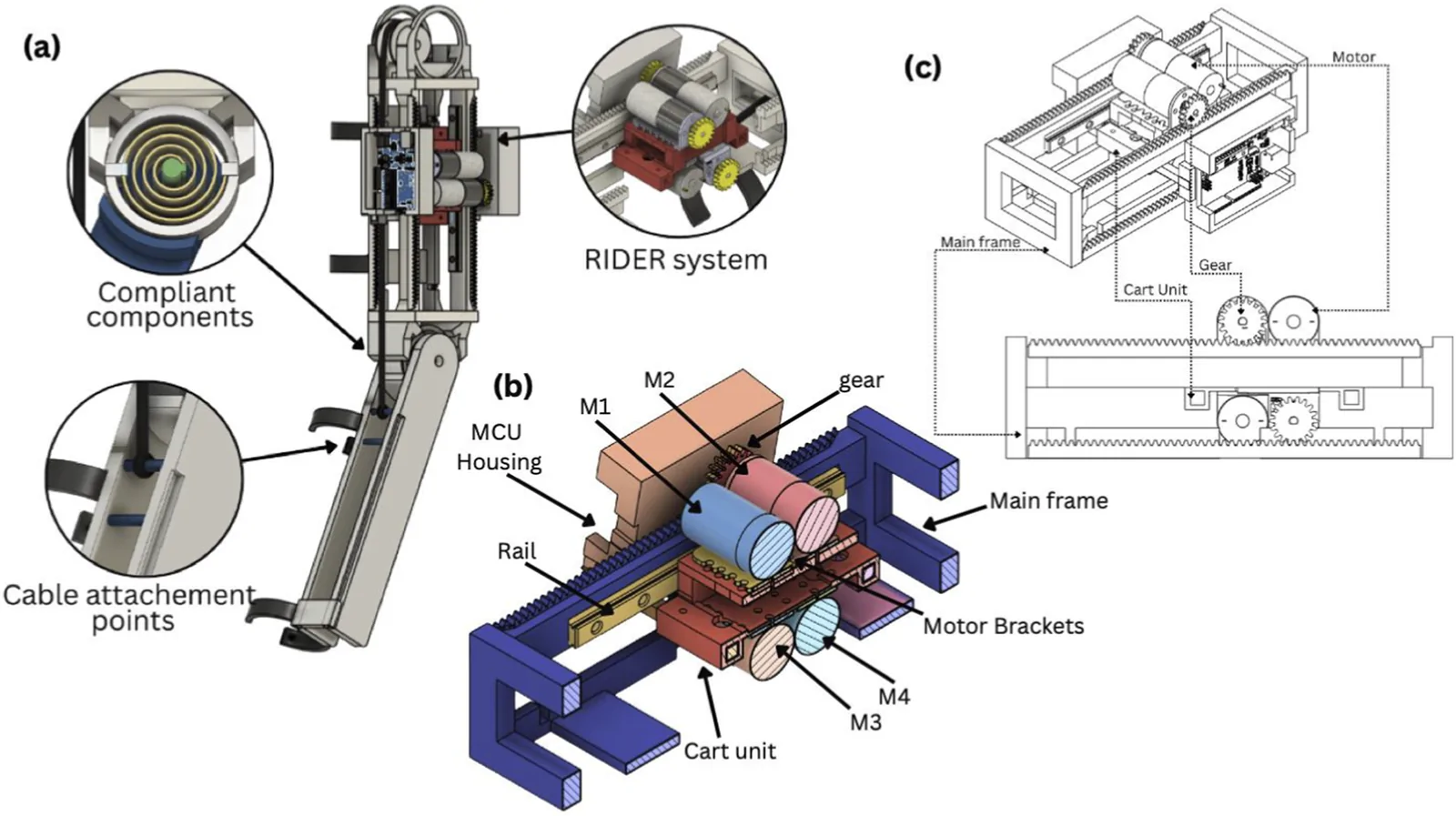
**(a)** Conceptual future elbow exoskeleton design with the RIDER system attached. **(b)** Cross-sectional view of the RIDER system. **(c)** Schematic of the RIDER system.

The RIDER system is composed of two primary mechanical subassemblies: (1) the main frame (Figure 2a) and (2) the cart unit (Figure 2b). The main frame serves as the structural support and housing of the rails and toothed track (Figure 2a), whereas the cart unit is mechanically constrained within the frame by the rail and only permitted to translate along the rail axis (Figure 3c). The linear motion of the cart relative to the main frame constitutes the actuation stroke of the system.

The motors mounted on the cart unit produce rotational output torque as the spur gears engage the toothed track on the main frame. As the gears rotate, tangential force is produced along the track, driving the cart unit longitudinally along the rail. This mechanism converts the motor’s rotational motion into controlled linear motion of the cart unit relative to the main frame, thereby generating linear actuation. A cable is attached to the cart unit to act as a “tendon” (Figure 3a). The cable is inelastic; thus, when the cart unit moves, the pulling force is directly transmitted through the cable, generating a joint torque.

The size of the system superstructure is 8 cm (width) × 6 cm (thickness) × 22 cm (height). With the protrusion of the two housing units on the sides, the width is 14.5 cm, and the two top motors increase the thickness to 8.5 cm.

#### 2.2.1 Actuator design

**FIGURE 4:**
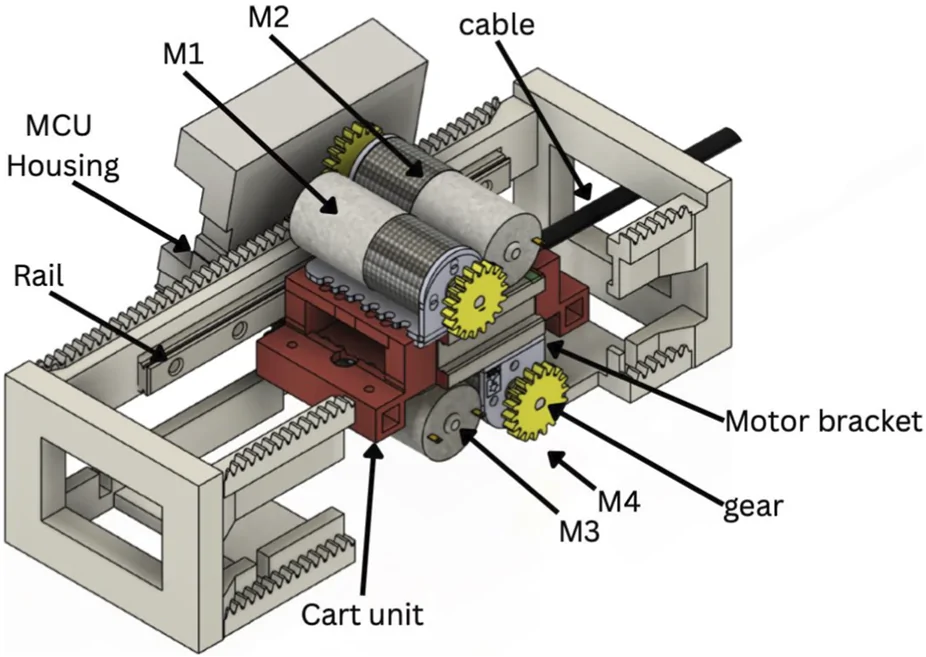
Layout of the RIDER system with parts labeled.

The RIDER system was modeled in Fusion (Autodesk, United States). The design is shown in Figure 2. The length of the actuator corresponds to the average male upper arm length as reported by Kudzia et al. (2022). The cart is driven by two 25D motors offset by 180°, which attach to the cart unit via the motor brackets. Notably, the layout allows for two additional motors (M3 and M4) (Figure 3b) to be added, further increasing the available linear pulling force of the actuator system (Figure 4).

An Arduino Uno microcontroller (MCU) is housed on the side of the cart unit to ensure proximity to the main motor driver (Figure 4) and to limit wire impingement and simplify cabling.

The MGN9 rails were implemented into the main frame for the cart to travel on (Figure 4). The rails stabilize the cart unit during travel and constrain it to one degree of freedom, controlling alignment relative to the toothed track system.

#### 2.2.2 Electrical design

**FIGURE 5:**
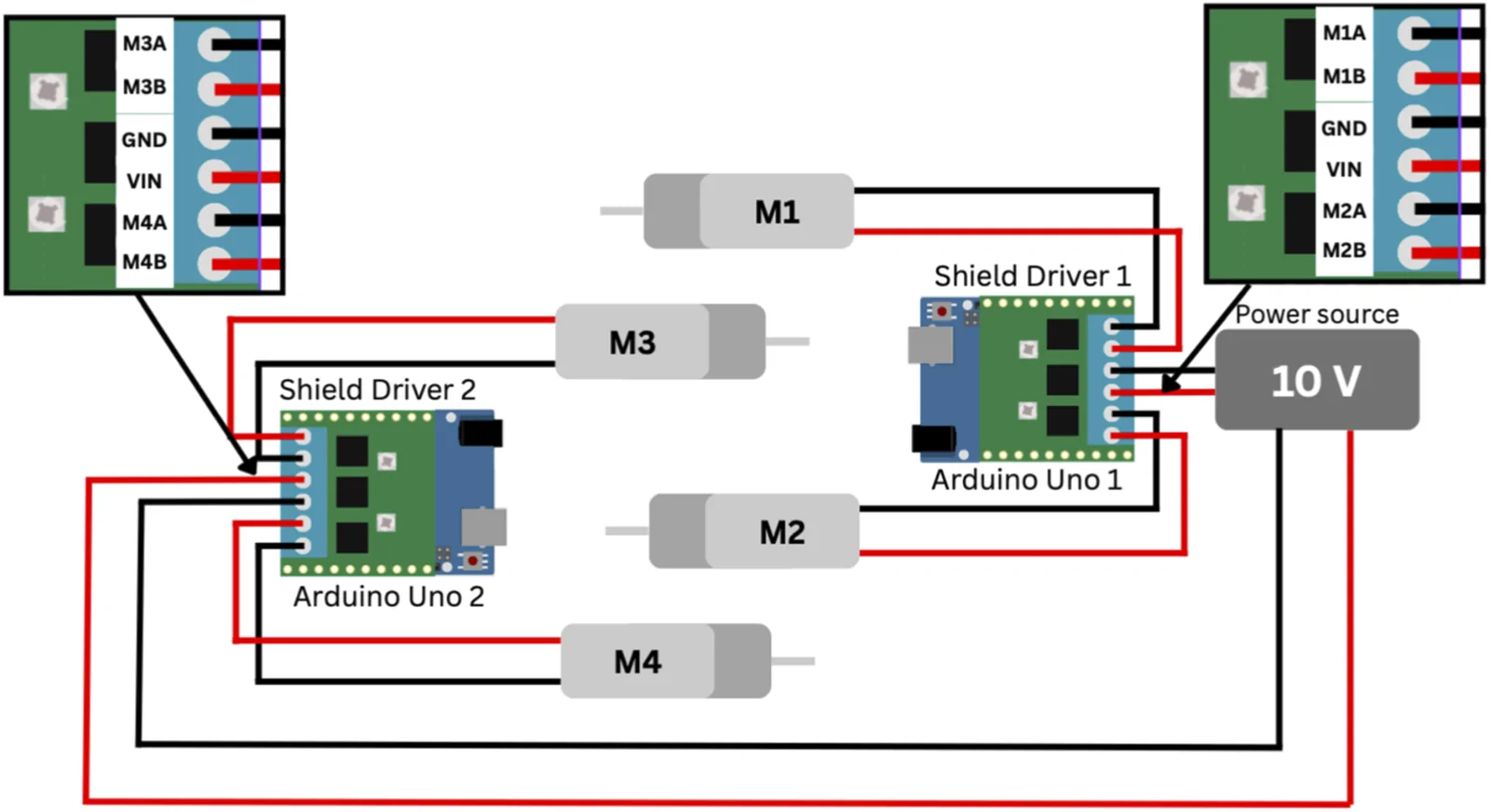
Wiring diagram of the motors to the motor shields. When assembling, the motor controllers are mounted directly onto the Arduino, and thus, a shield driver image is depicted here to simplify the image.

To actuate the system, we use four 12V, 79 RPM DC motors (Pololu Corporation, 25D Metal Gearmotor 12V 100:1 HP, United States). Each motor has a stall torque of 1.079 Nm (Pololu, 2025a). We chose this motor for its compatible size (25 D × 54 L mm) and torque output (Pololu, 2025a). The motors were regulated by Pololu Motor driver shields (Pololu Corporation, Dual MC33926 Motor Driver Shield for Arduino, United States) (Pololu, 2025b). The motor driver shield was connected to the Arduino Uno to regulate the motors. The motor shields were powered directly by a 10 V power supply, as shown in Figure 5.

**FIGURE 6:**
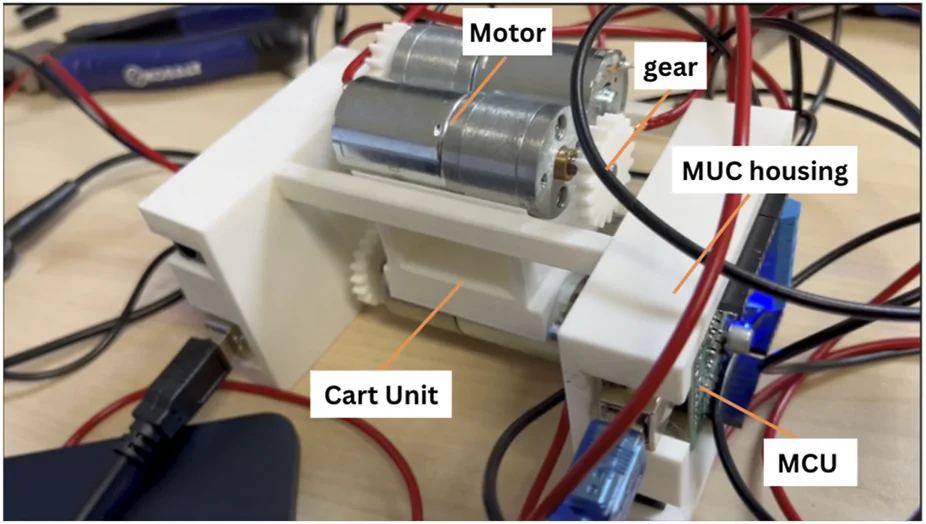
Mock-up image of electrical components on the cart unit.

Figure 5 illustrates the wiring configuration of the motor shield assembly. There are six ports on each shield. The M1A and M1B ports on shield driver 1 connect to the ground and power of the first motor, M1, respectively, supplying it with a signal and powering it. The GND and VIN ports were connected to the power supply. Similarly, the bottom M2A and M2B ports power and control the second motor, M2. The second set of motors, M3 and M4, are connected to shield driver 2 on the Arduino in the same manner. Each motor can then be controlled separately with this configuration. The configuration of the fully assembled cart unit with both sets of motor shields is shown in Figure 6. The shield drivers are mounted directly on the Arduino Uno boards, which can connect to a single driver. Thus, two Arduino units were used to control four motors in the RIDER system. Future implementations can simplify this by using motor drivers that control four motors or selecting a microcontroller that can connect to multiple drivers.

The RIDER system utilizes a distributed leader–listener control architecture. The leader MCU detects user input and generates the related motion command outputs for a single motor group. The listener MCU mirrors these motion commands generated by the leader MCU and controls the other motor group. This ensures that both sets of motor groups operate in unison during translational operation of the cart unit, enabling coordinated directional actuation.

#### 2.2.3 System torque design and requirements

For functional use, we require to produce torques in the same range as a healthy individual, as reported by Kudzia et al. (2022) and Jenks et al. (2025). The torque, $\tau_{y},$ of a normal human individual can be calculated using Equation 1, where $F_{g}$ represents the weight of the forearm/hand and l is the distance between the center of mass (COM) of the forearm and the elbow joint. Thus, the required torque, $\tau_{y}$ , for the actuator system, is at least 4.75 Nm, as reported in a previous study (Jenks et al., 2025):

$$\tau_{y}=F_{g}*l.$$ (1)

Future use of this system will be in assistive rehabilitation exoskeletons, and it must provide torque assistance across healthy to paretic states. Since the loss in torque of a paretic individual relative to a healthy individual is approximately 47% (Equation 2), we determine the torque loss, $\tau_{lost}$ , as approximately 2.991 Nm (Jenks et al., 2025). Therefore, the functional torque should be at least in this range:

$$\tau_{lost}=0.63\tau_{y}.$$ (2)

Theoretical torque output and achievable joint range of motion (ROM) were estimated based on the actuator force output capability and stroke length. Here, the torque output was determined using Equation 3, where τ is the joint torque output, *F* is the linear cable pulling force supplied by the actuator, and *r* is the distance between the cable attachment point and the elbow joint (effective moment arm):

τ=F·r. (3)

**FIGURE 7:**
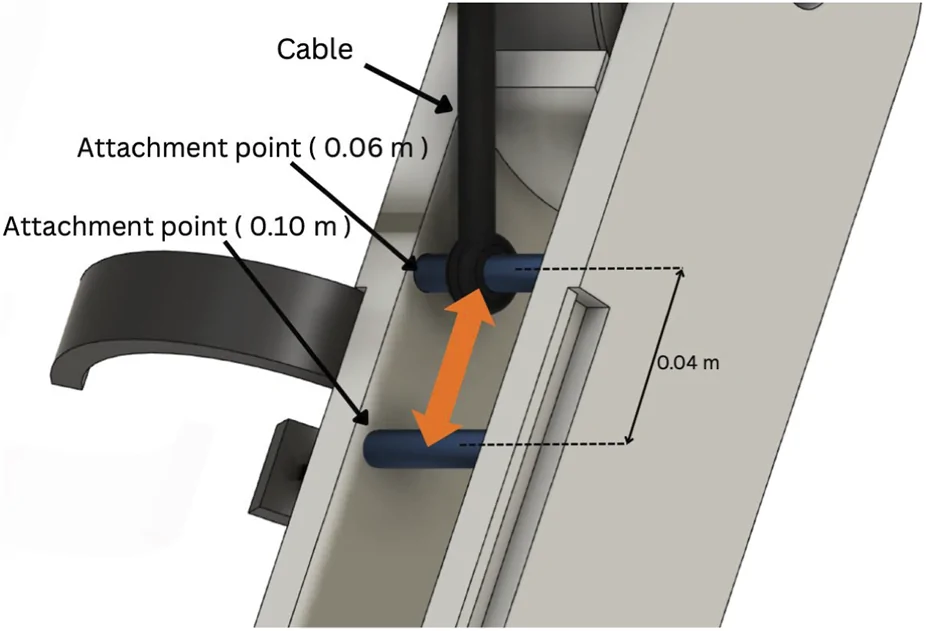
Example cable attachment points for calculations on future exoskeleton segments.

Our future exoskeleton will have two attachment points for two operational modes (Figure 7), and thus, we calculate torque under two conditions:

1. Performance augmentation mode: r = 0.10 m
2. Rehabilitation mode: r = 0.06 m

The performance augmentation mode has a larger effective moment arm, r, based on the cable attachment position (Figure 7)—e.g., r = 0.10 m instead of r = 0.06 m. This increases the torque output but reduces achievable ROM as a trade-off. Conversely, the rehabilitation mode has a shorter moment arm (r), thus decreasing torque output, but a larger ROM. For ROM estimation, the relationship between linear cable displacement and joint rotation was calculated using Equation 4, where ΔL is the cable travel displacement (stroke length) and Δϕ is the elbow joint angular displacement:

$$\Delta \phi =\frac{\Delta L}{r}.$$ (4)

### 2.3 Manufacturing and assembly

**FIGURE 8:**
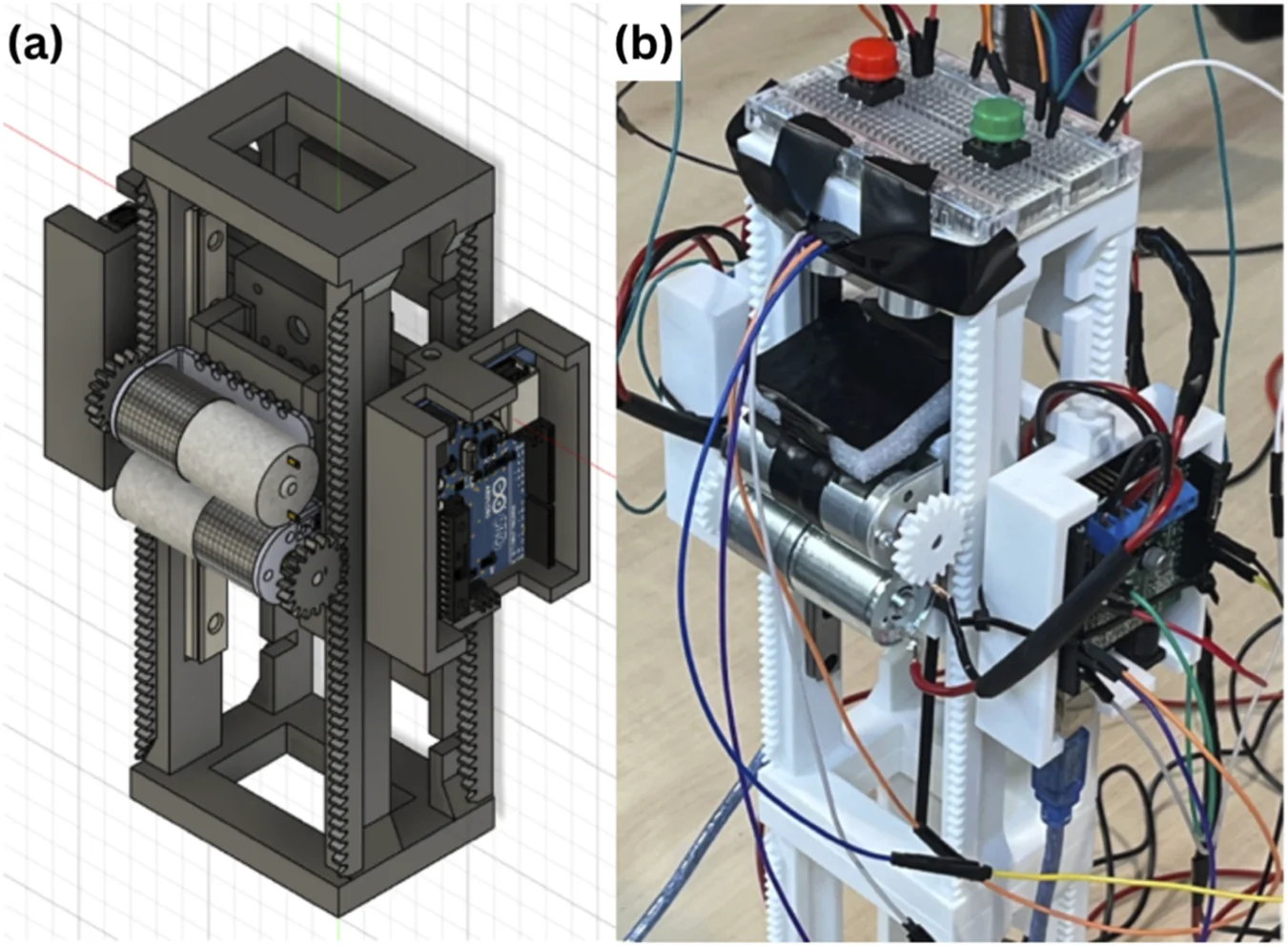
**(a)**CAD design of the RIDER system. **(b)**Fully assembled RIDER system prototype during testing.

Figure 8 shows the fully assembled RIDER system and the Fusion design side by side. The system was assembled by attaching the two rails to the inside of the main frame and firmly pressing them into the slots on the two sides of the inner walls. Next, the cart unit was placed into the main frame, with the rails attached on the inside. The cart unit was placed between the rails and attached to the four motor brackets with screws. This firmly secured the cart unit to the moving platform of the rails. Then, the motors were mounted to the cart unit brackets using screws. Finally, the Arduino was added to the left and right unit housings and placed into two rectangular slots on the sides of the cart unit.

The components, such as the rails and motor bracket, are commercially available, whereas the main frame, cart units, and gears were 3D-printed in PLA. PLA was chosen because of its low density and sufficient mechanical strength for prototyping purposes. The main frame and the cart system were printed with 25% infill using a triangular grid pattern. This infill percentage is deemed appropriate for linear loads since the system is not subject to considerable torsional effects. The gears were 3D-printed with 100% infill to maximize their strength.

### 2.4 Experimental setup

To evaluate the performance of the RIDER system, we conducted a force–velocity characterization test to determine the maximum force output and operational speed using controlled loading under constant voltage (Firgelli Automations Team, 2020). The force and velocity provide information on the exoskeleton’s operational limits and speed under various conditions.

**FIGURE 9:**
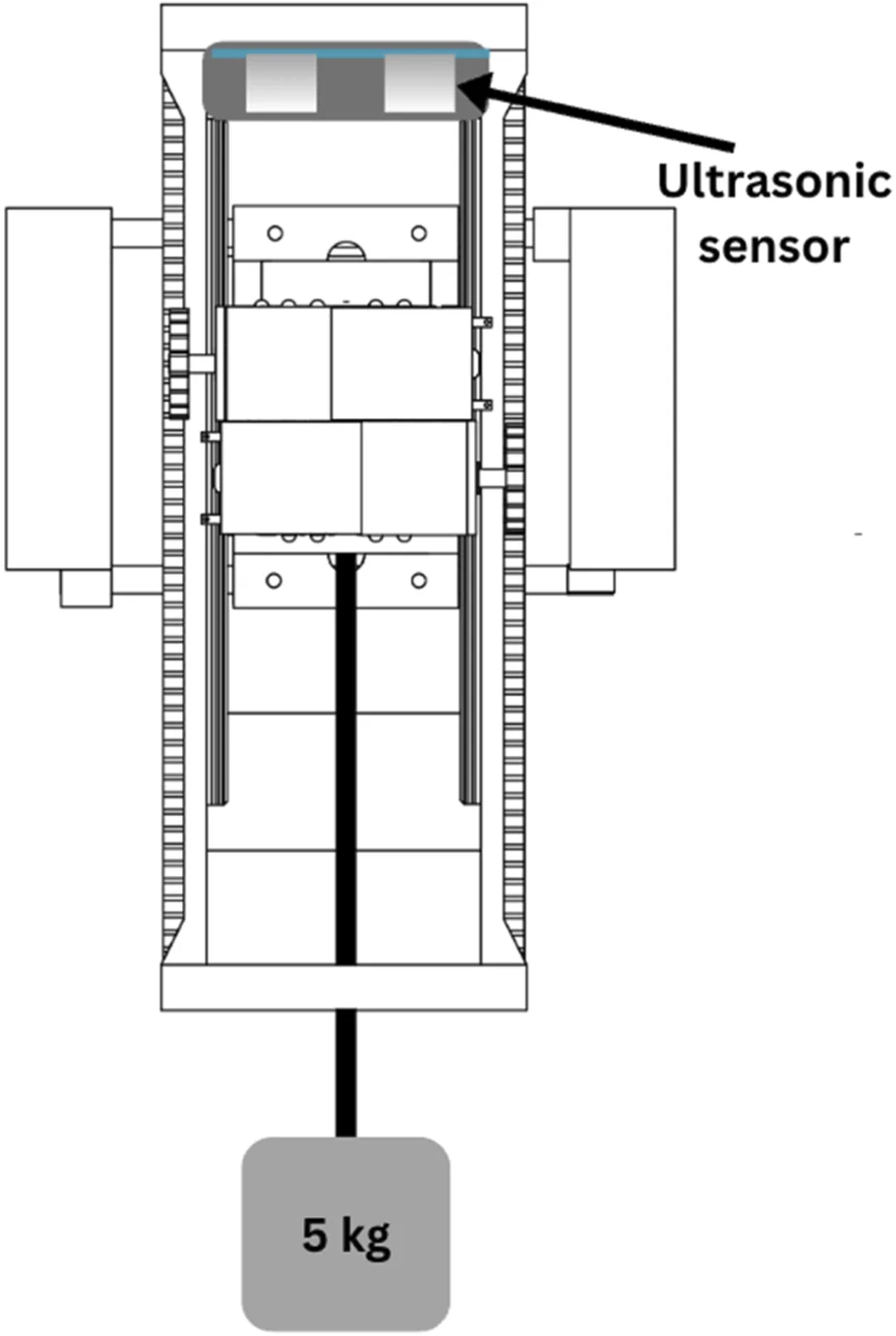
Representation of the experimental setup.

An ultrasonic sensor (HC-SR04) was mounted at the top end of the track within the main frame to measure the real-time displacement of the cart system during operation (Figure 9). The sensor has an operational range of 2–400 cm and a ranging accuracy of 3 mm. The sensor is placed so that the relative operational range of the actuator is within 4–12 cm of the sensor and thus in its functional range. The RIDER system was vertically oriented as if worn on the body, and the cart unit was connected to an external mass. During testing, the cart unit moved linearly to lift the attached mass against gravity. Increasingly heavy masses were used to evaluate the dynamic response. Specifically, the weights were chosen based on standard imperial sizes: 2.5 lbs (1,145 g), 5 lbs (2,269 g), 7.5 lbs (3,404 g), 10 lbs (4,543 g), 15 lbs (6,746 g), 20 lbs (9,019 g), and 22.5 lbs (1,0571 g). The weight of the attached mass was compared with the velocity of the cart unit to determine the force–velocity relationship. Each weight-movement task was repeated five times to obtain the repeatable performance. However, for weights that approached system stall force were repeated only three times to preserve the service life of the motors.

With this experiment, two limiting cases were evaluated: (1) the maximum load and (2) no-load conditions. The maximum load condition shows the stall force and corresponds to the RIDER system’s maximum force. In other words, the weight of the attached mass affected the velocity of the cart unit and slowed translation to near zero. The freeload condition measured the actuator’s velocity (i.e., maximum speed) without a load. Here, no external mass was attached to the cart unit.

The velocity was calculated from the cart’s displacement measured by the ultrasonic sensor. Distance data were first smoothed using a moving-average filter to reduce noise. Then, the velocity was calculated by numerical differentiation of the position relative to the Arduino clock.

#### 2.4.1 Software control

For the experimental protocol, the motors were operated in an open loop with pulse-width modulation (PWM) at a duty cycle of 255/255 (maximum speed). They were powered with 10 V from a power supply driven by the motor shield. The full code is available on the provided GitHub link.

## 3 Results

**FIGURE 10:**
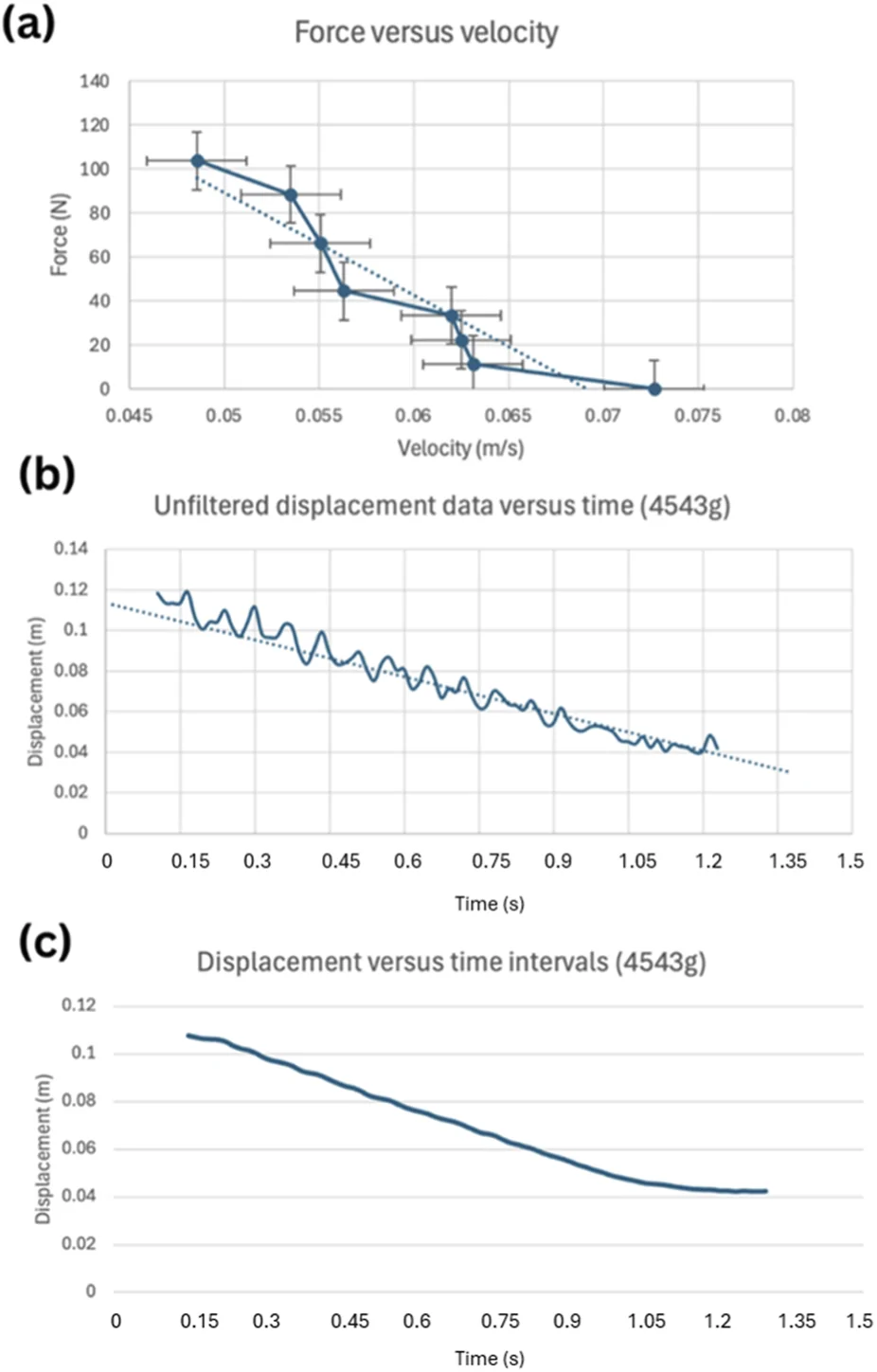
**(a)**Measured force–velocity relationship of the RIDER system, **(b)**unfiltered displacement data versus time, and **(c)**filtered displacement versus time at mass 4,543 g.

In total, eight trials were conducted for this test using weights from 1,145 to 10,571 g. Figure 10a shows the force–velocity relationship of the RIDER system with the corresponding data summarized in Table 1. The velocity decreased from 6.31 to 4.86 cm/s with an increase in the applied load from 1,145 to 10,571 g. The freeload velocity was measured at 7.3 cm/s, and the maximum tested load was 103.7 N.

**TABLE 1:** The force and velocity testing data of the RIDER system.

Mass (g)	Force (N)	Mean velocity (cm/s)	Max (cm/s)	Min (cm/s)
0	0	7.27 ± 0.23	7.60	7.06
1,145	11.2	6.31 ± 0.26	6.65	6.06
2,269	22.3	6.25 ± 0.32	6.73	5.95
3,404	33.4	6.20 ± 0.15	6.25	5.91
4,543	44.6	5.64 ± 0.25	6.02	5.33
6,746	66.2	5.51 ± 0.16	5.67	5.27
9,019	88.5	5.35 ± 1.33	6.43	3.78
10,571	103.7	4.86 ± 1.03	5.51	5.36

The maximum tested load represents the RIDER system’s highest achievable force output. Figure 10b shows representative displacement data sampled under a midrange load condition (4,543 g). The figure shows that the cart exhibits approximately linear translational behavior over time. The displacement data were smoothed utilizing a moving average filter with outliers removed prior to velocity calculation (Figure 10b).

The force-to-mass ratio was calculated to be 105 N/kg. Using the maximum force, 103.7 N, the theoretical elbow torque was estimated (Equation 3) to be 6.22 Nm (rehabilitation mode, r = 0.06 m) and 10.37 Nm (performance augmentation mode, r = 0.10 m). Based on the measured stroke length of 0.125 m (Table 2), the theoretical maximum angular displacement was estimated using Equation 4. For the rehabilitation mode, the angular displacement was estimated to be 120°, and for the performance augmentation mode, it was 72° (Table 3).

**TABLE 2:** Geometric and mechanical performance of the RIDER system.

Characteristic	Values
Maximum cart speed (cm/s)	7.27 ± 0.23
Maximum force (N)	103.7
Actuator mass (kg)	0.99
Stroke length (cm)	12.5
Actuator length (cm)	21.7
Operating voltage (V)	10

**TABLE 3:** Theoretical elbow torque and ROM for the two operation modes.

Modes	Moment arm (m)	Torque (Nm)	Angular displacement
Rehabilitation	0.06	6.22	72°
Augmentation	0.10	10.37	120°

As shown in Table 3, due to the configuration differences between the two modes, increasing the moment arm increases the output torque but decreases the angular displacement.

## 4 Discussion

Our work presents an integrated rail-based linear actuator designed for tendon-driven elbow exoskeletons. This device was mechanically tested to determine its functional metrics for future tendon-driven exoskeletons. It was demonstrated that the RIDER system could achieve a torque output of 10.37 Nm in the performance augmentation mode. Similarly, in the rehabilitation mode, the output torque was determined to be 6.22 Nm. In both cases, the torque exceeds the assistance torque needed for an exoskeleton (4.75 Nm) and thus meets the performance requirements for our future studies that integrate the RIDER system into a poststroke rehabilitation exoskeleton. The RIDER system was conceptually designed for longitudinal placement along the upper arm to minimize any potential interference during flexion and extension. The self-contained rail-based configuration was intended to distribute the actuator mass along the entire arm segment rather than concentrating it at the joint. The tendon-driven cable routing provides flexibility in actuator placement, thereby reducing mechanical obstruction around the elbow joint. The actuator layout is symmetrical, allowing the same configuration to be mirrored for integration on either arm. This bilateral compatibility may simplify future wearable implementation and improve convenience across different configurations.

### 4.1 Actuator performance and mechanical limitation

The force–velocity relationship in Figure 10a demonstrates that the RIDER system exhibits loading characteristics consistent with those of conventional linear actuators. However, the maximum force output in this study, 103.7 N, was determined by the engagement failure point (loss of force transmission through rails) rather than by motor stall torque. The engagement failure occurs when the gears lose traction on the track under excessive load, rotating but not maintaining full traction. This prevents further upward translation while maintaining positional stability, keeping the cart unit supported at the achieved height without experiencing downward motion or uncontrolled back driving. Notably, structure derailment does not occur due to the integrated rail guide system. Future work will also explore how this changes with the number of active motors to scale stall or holding performance of the system.

Table 4 compares the geometric and mechanical performance of the RIDER system with a representative commercial telescopic linear actuator. The comparison was selected since this linear actuator exhibits a similar overall geometric footprint. As shown in the table, the RIDER system has a higher stroke-to-length ratio than this commercial telescopic actuator (0.58 vs. 0.20). However, the force output of the RIDER system is somewhat lower. Although the telescopic linear actuator might provide higher force output, its limited stroke-to-length ratio limits achievable joint rotation when integrated into an exoskeleton. Similarly, geometric integration is more difficult in a constrained wearable environment. This spatial efficiency is especially relevant in upper-limb exoskeleton design, where the available mounting space is limited.

**TABLE 4:** Comparison between the RIDER system and a representative commercial telescopic linear actuator.

Parameters	RIDER system	Telescopic linear actuator
Maximum speed (cm/s)	7.27 ± 0.23	5.08
Maximum tested force (N)	103	156
Actuator weight (N)	9.73	12
Strength-to-mass ratio (N/kg)	103.8	127.5
Stroke length (cm)	12.5	5
Total length (cm)	21.7	20.1–25.2
Stroke-to-length ratio	0.58	0.20

As shown in Table 4, the RIDER system achieves a stroke length of 12.5 cm with an actuator length of 21.7 cm. This results in a stroke-to-length ratio of 0.58, which is significantly higher than that of the traditional telescopic linear actuator used for comparison. Conveniently, the mass of the RIDER system is longitudinally distributed along the upper arm and improves wearability (Perry et al., 2007). In total, RIDER only weighs 9.73 N with all components included, allowing it to be integrated into a wearable configuration and eliminating the need for external platforms.

Here we compare the compactness and stroke-to-length ratio with actuators used in other systems. The hydraulic actuator model chosen for comparison was a commercial round-body hydraulic cylinder (Round Body Hydraulic Cylinder - 20mm Bore, 75 mm Stroke - Pascal Corporation, Japan) as it exhibits a similar overall geometric footprint and is representative of hydraulic linear actuators used in arm exoskeletons. The retracted length serves as the dimensional metric for comparing the compactness of the actuators for integration. The stroke length is intended to demonstrate the actuator’s extension capability. As shown in Table 5, to compare the actuator extension, the RIDER system was compared with the hydraulic and electrical actuators of similar retracted lengths. The RIDER system achieves the highest stroke length among actuators of comparable size, resulting in the highest stroke-to-length ratio (0.58) when compared with representative hydraulic (0.35) and electrical (0.20) linear actuators.

**TABLE 5:** Comparison of stroke length between the RIDER system and representative commercial linear actuators with similar retracted length.

Actuators	Stroke length (cm)	Retracted length (cm)	Stroke-to-length ratio
Hydraulic	7.5	21.3	0.35
Electrical	5	20.1	0.20
RIDER	12.5	21.7	0.58

As shown in Table 6, to compare the actuator compactness for integration, the RIDER system was compared with hydraulic and electrical actuators of similar stroke lengths. The RIDER system achieves the shortest retracted length among actuators of comparable stroke lengths, indicating improved compact integration for wearable applications.

**TABLE 6:** Comparison of retracted length between the RIDER system and representative commercial linear actuators with stroke length.

Actuators	Stroke length (cm)	Retracted length (cm)	Stroke-to-length ratio
Hydraulic	12.5	26.3	0.35
Electrical	15.2	30.2	0.20
RIDER	12.5	21.7	0.58

**TABLE 7:** Cost analysis of the RIDER system prototype.

Item	Quantity	Cost (USD)
Arduino Uno	×2	$20
25D motor	×4	$33.95
Driver shield	×2	$31.95
Motor bracket	×4	$10.95
MGN9 rails	×2	$12.88
PLA	N/A	∼$30
Total	N/A	$339.26

The total material cost of the RIDER system prototype is listed in Table 7. This includes the motors, MCUs, motor drivers, linear rails, and the approximate 3D printing material cost.

### 4.2 Dual-mode torque–ROM analysis

Table 3 illustrates the trade-off between torque amplification and angular displacement in a dual-mode configuration. This function enables adaptability to specific tasks in future experimentation. For example, the rehabilitation configuration allows greater ROM (120°) and approaches the natural human elbow ROM of 130° (Zwerus et al., 2019). Conversely, the performance augmentation has a ROM of 72° but a higher torque.

From a torque perspective, the rehabilitation configuration generates a torque output of 6.22 Nm, and performance augmentation provides a torque of 10.37 Nm. In both cases, the torque outputs exceed the requirement for poststroke movement assistance. This demonstrates that the RIDER system is capable of generating enough torque as needed for an assistive exoskeleton.

### 4.3 Current limitations

This study is limited to benchtop testing of the RIDER system and actuator characterization. The reported torque and ROM are theoretical estimates calculated from the measured torque output and extension stroke length and do not reflect real experimental joint-assistance performance. In addition, the prototype uses a simplified open-loop PWM to move the motor, rather than complex joint biomechanics for use in an exoskeleton. This simplified control setup is not intended for the final wearable operation. For future real-world human–robot interaction studies, the RIDER system will feature a closed-loop position and force control to ensure the safety and repeatability of assistive operations. Furthermore, in practical wearable operation, elbow assistance would require dynamic torque output that varies throughout flexion and extension, rather than constant loading as tested in the current paradigm. Regardless, when comparing loading characteristics with other commercially available actuators, the RIDER system demonstrates a higher stroke-to-length ratio while maintaining a similar force-to-mass ratio.

Additionally, the RIDER prototype is fabricated from PLA 3D-printed components. During benchtop testing, the gear and rail interface experienced the highest localized mechanical stress and visible warping during repeated contact loading and sliding. In future versions, stronger materials for the gear–rail interface will be implemented to withstand repetitive cyclic operations during real-world operations and assistive applications. Similarly, scalability to higher torque outputs and long-term wearable operation will also require stronger structural materials than those used in the current PLA benchtop prototype. Furthermore, the current system operates without integrated force feedback, joint position sensing, or adaptive human-interaction control algorithms. While this is sufficient for mechanical actuator characterization, future work will include closed-loop sensing and dynamic control strategies.

### 4.4 Finite element analysis

**FIGURE 11:**
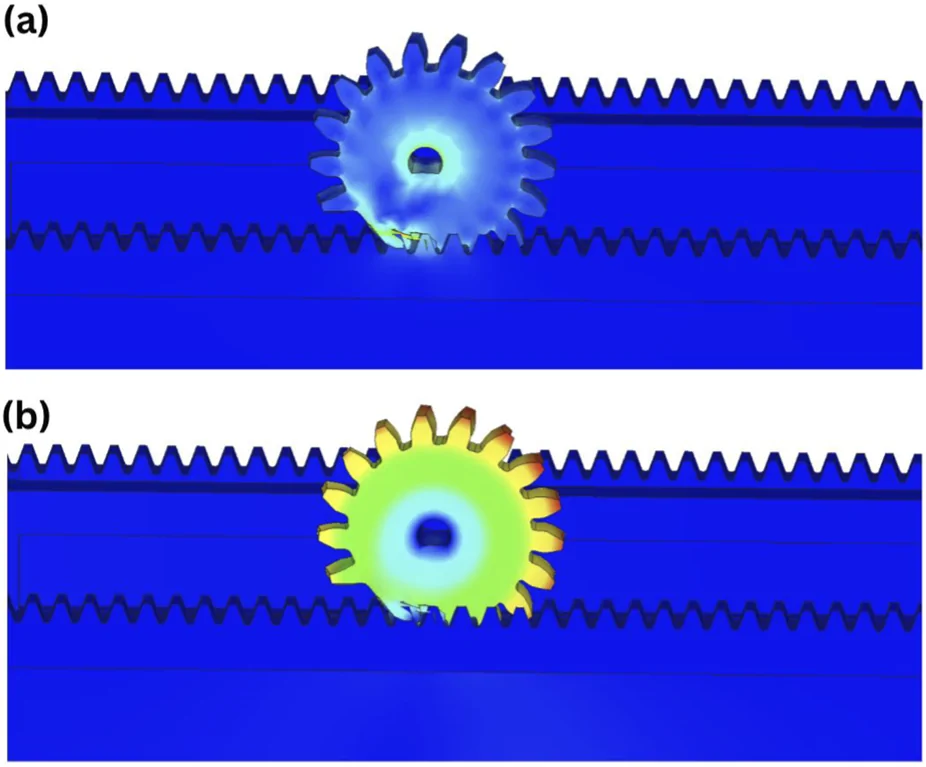
**(a)** Stress and **(b)** displacement profiles of the gear design under rotation load.

A finite element analysis (FEA) of the gear–track interaction was performed. During the experimental condition, wear occurred on the gear, so the analysis was intended to identify the stress concentration region (Figure 11a) and deformation (Figure 11b). FEA shows a maximum Von Mises stress of 39.8 MPa located at the gear–rail interface and matches the wear location on the gear during actual experimentation. This corresponds to a maximum deformation of 0.051 mm when using a 1 Nm applied torque as a conservative loading condition.

### 4.5 Cost analysis

## 5 Conclusion

This study developed the RIDER system, a rail-based linear actuator for tendon-driven upper-limb exoskeletons. The modularity of the design enables for utility in two modes: elbow performance augmentation and rehabilitation. For example, the performance augmentation mode provides an estimated torque output of 10.37 Nm while allowing approximately a ROM of 72°. The rehabilitation mode delivers an estimated torque of 6.22 Nm while allowing an elbow joint ROM of 120°. This system allows for a controllable trade-off between torque amplification and ROM for specific task applications. In addition, the RIDER system addresses some limitations of traditional linear actuators such as the limited stroke-to-length ratio and mechanical bulk. Force–velocity testing demonstrated load-dependent performance expected for a linear actuator, with a maximum tested load of 103.7 N.

Compared with a representative telescopic linear actuator of similar size, the RIDER system allows for a higher stroke-to-length ratio, enabling improved geometric integration along the upper arm segment. While gear–track engagement has some limitations, the configuration does limit back driving. In future studies, we will investigate how geometries can be optimized to scale torque output and ROM and to employ stronger materials (e.g., carbon fiber or metal) for real-world applications. Our future study will examine the effectiveness of a RIDER actuation system in assistive exoskeletons for poststroke neurorehabilitation.

## Data Availability

The original contributions presented in the study are included in the article/Supplementary Material; further inquiries can be directed to the corresponding author.
